# A Response Surface Methodology Approach to Investigate the Effect of Sulfur Dioxide, pH, and Ethanol on *DbCD* and *DbVPR* Gene Expression and on the Volatile Phenol Production in *Dekkera/Brettanomyces bruxellensis CBS2499*

**DOI:** 10.3389/fmicb.2017.01727

**Published:** 2017-09-11

**Authors:** Federica Valdetara, Daniela Fracassetti, Alessia Campanello, Carlo Costa, Roberto Foschino, Concetta Compagno, Ileana Vigentini

**Affiliations:** ^1^Department of Food, Environmental and Nutritional Sciences, Università degli Studi di Milano Milan, Italy; ^2^Eppendorf s.r.l. Milan, Italy

**Keywords:** *D./B. bruxellensis*, volatile phenols, cinnamate decarboxylase gene, vinylphenol reductase gene, gene expression, response surface methodology

## Abstract

*Dekkera/Brettanomyces bruxellensis*, the main spoilage yeast in barrel-aged wine, metabolize hydroxycinnamic acids into off-flavors, namely ethylphenols. Recently, both the enzymes involved in this transformation, the cinnamate decarboxylase (*DbCD*) and the vinylphenol reductase (*DbVPR*), have been identified. To counteract microbial proliferation in wine, sulfur dioxide (SO_2_) is used commonly to stabilize the final product, but limiting its use is advised to preserve human health and boost sustainability in winemaking. In the present study, the influence of SO_2_ was investigated in relation with pH and ethanol factors on the expression of *DbCD* and *DbVPR* genes and volatile phenol production in *D. bruxellensis* CBS2499 strain under different model wines throughout a response surface methodology (RSM). In order to ensure an exact quantification of *DbCD* and *DbVPR* expression, an appropriate housekeeping gene was sought among *DbPDC*, *DbALD*, *DbEF*, *DbACT*, and *DbTUB* genes by GeNorm and Normfinder algorithms. The latter gene showed the highest expression stability and it was chosen as the reference housekeeping gene in qPCR assays. Even though SO_2_ could not be commented as main factor because of its statistical irrelevance on the response of *DbCD* gene, linear interactions with pH and ethanol concurred to define a significant effect (*p* < 0.05) on its expression. The *DbCD* gene was generally downregulated respect to a permissive growth condition (0 mg/L mol. SO_2_, pH 4.5 and 5% v/v ethanol); the combination of the factor levels that maximizes its expression (0.83-fold change) was calculated at 0.25 mg/L mol. SO_2_, pH 4.5 and 12.5% (v/v) ethanol. On the contrary, *DbVPR* expression was not influenced by main factors or by their interactions; however, its expression is maximized (1.80-fold change) at the same conditions calculated for *DbCD* gene. While no linear interaction between factors influenced the off-flavor synthesis, ethanol and pH produced a significant effect as individual factors. The obtained results can be useful to improve the SO_2_ management at the grape harvesting and during winemaking in order to minimize the *D./B. bruxellensis* spoilage.

## Introduction

During the aging of red wines, mainly if they are stored in barrels, undesirable metabolites (off-flavors) can appear due to the growth of contaminating yeasts, such as *Dekkera/Brettanomyces bruxellensis* species (Silva et al., 2004). This sensory modification resulting in wine defect is termed “Brett character” and it is described by “leather,” “horse sweat,” “medicinal,” “barnyard,” and “bacon” descriptors (Chatonnet et al., 1995). In general, the spoilage by *Dekkera/Brettanomyces* yeasts can causes huge economic loss in wine industry and several methods for its rapid detection has been proposed (Tofalo et al., 2012; Vigentini et al., 2012; Uusitalo et al., 2017).

The origin of volatile phenols (VPs) involves the sequential action of enzymes acting on hydroxycinnamic acids, substrates that can be obtained through the activity of cinnamoyl-esterase enzyme on their respective cinnamic acids or released by fungal enzymes or by grape juice heating (Gerbaux et al., 2002). Being toxic for many microorganisms, hydroxycinnamic acids are decarboxylated by the action of cinnamate decarboxylase (CD), thus allowing a detoxification of the environment (Edlin et al., 1998).

It has been reported that the activity of CD releases vinyl derivatives (4-vinylphenol, 4-vinylguaiacol, and 4-vinylcatechol) (Dias et al., 2003a; Edlin et al., 1995). In particular, in *B. bruxellensis* LAMAP2480 a CD was identified as phenylacrylic acid decarboxylase (PAD1p), which is responsible for the production of 4-vinylphenol from *p*-coumaric acid, and encoded by the corresponding *DbPAD* gene (Godoy et al., 2014). Vinyl phenols are reduced into their corresponding ethyl derivatives (4-ethylphenol, 4-ethylguaiacol, and 4-ethylcatechol) in a step catalyzed by a vinylphenol reductase (VPR) that represents the key enzyme designating *D./B. bruxellensis* species as the spoilage yeast able to produce ethyl phenols. VPR enzyme was identified in *D. bruxellensis* CBS4481 as a Zn/Cu superoxide dismutase (SOD1) belonging a NAD(P)H-dependent oxidoreductases of the Short-chain Dehydrogenases/Reductases (SDRs) family (Granato et al., 2014). The cloning of *DbVPR* gene in *Saccharomyces cerevisiae*, a species not producing ethyl phenols, has recently confirmed its role in the off-flavor production (Romano et al., 2017).

The concentration of some wine components (i.e., ethanol, sugars, and VPs) and some chemical factors (i.e., pH and sulfur dioxide) have been demonstrated affecting the occurrence of off-flavors by *D./B. bruxellensis* (Dias et al., 2003b; Godoy et al., 2008; Sturm et al., 2014). This evidence has posed the need to investigate the interaction among multiple aspects on the production of VPs (Ganga et al., 2011; Chandra et al., 2014). For example, the influence of interactions due to the presence of *p*-coumaric acid, ferulic acid, and ethanol on CD activity and the expression of its putative gene has been studied (Ganga et al., 2011). Results outlined that although oenological concentrations of *p*-coumaric and ferulic acids alone did not produced any significant effect on the enzyme activity, this was influenced by interactions between ethanol and cinnamic acid or temperature. Recently, Chandra et al. (2014) analyzed the effect of glucose, ethanol and SO_2_ on the growth and VP production by *B. bruxellensis* ISA 2211. A negative linear and quadratic effect triggered by SO_2_ occurred on growth and 4-ethylphenol production; in particular, a SO_2_ concentrations higher than 20 mg/L, at pH 3.50, induced immediate loss of cell culturability even under growth permissive levels of ethanol.

“Bret” character is often associated to the capability of *Brettanomyces* yeasts to grow under low level of molecular SO_2_ concentration (Barata et al., 2008; Curtin C. et al., 2012; Vigentini et al., 2013). Thus, using high concentrations of SO_2_ could ensure failure of *Brettanomyces* spoilage. However, reducing sulfite in wine represents a valuable task in view of a sustainable implementation in winemaking and a better acceptability for the consumers’ health. The present study has investigated the expression of *DbCD* and *DbVPR* genes, being recently identified with certainty (Godoy et al., 2014; Romano et al., 2017), and the production of VPs in relation with wine’s factors as SO_2_, pH, and ethanol throughout a response surface methodology (RSM). The choice of the factors ensued taking into consideration that molecular SO_2_ concentration depends on pH, ethanol concentration, and temperature (Usseglio-Tomasset and Bosia, 1984; Ribéreau-Gayon et al., 2006) and that, the latter is possibly the only manageable factor in aging process. Moreover, in order to ensure an exact quantification of mRNA transcription profile of *DbCD* and *DbVPR*, in the condition under study, an appropriate housekeeping gene (HKG) was identified.

## Materials and Methods

### Yeast Strain and Maintenance

*Dekkera bruxellensis* CBS2499 was used in this study. Its whole genome sequence is available at http://genome.jgi.doe.gov/Dekbr2/Dekbr2.home.html (Piškur et al., 2012). Cells were stored in YPD medium (10 g/L yeast extract, 20 g/L peptone, 20 g/L glucose, 5.5 pH) supplemented with 20% (v/v) glycerol at -80°C. Cell revitalization was performed inoculating the glycerol stock at 1% (v/v) in YPD broth. Cultures were placed into an incubator (Heidolph, Schwabach, Germany) at 30°C for 3 days.

### Growth Media and Culture Conditions

Experiments were run to collect yeast biomass for RNA extraction, retrotranscription and the analysis of gene expression by real-time quantitative PCR (qPCR). All fermentations were carried out in simil-wine Medium (SWM) [2.50 g/L glucose, 2.50 g/L fructose, 5 g/L glycerol, 5 g/L tartaric acid, 0.50 g/L malic acid, 0.20 g/L citric acid, 4 g/L L-lactic acid, 1.70 g/L yeast nitrogen base w/o AA and ammonium sulfate (Difco, Sparks, MD, United States), 0.005 g/L oleic acid, 0.50 mL tween 80, 0.015 g/L ergosterol, 0.020 g/L uracil, 0.010 g/L *p*-coumaric, 0.010 g/L ferulic acid, and 1.50 g/L ammonium sulfate]. Variants of SWM were prepared at different molecular SO_2_ (below: SO_2_) and ethanol concentration and pH value, adjusted with NaOH, depending on the conditions set by the chosen RSM (**Table 1**). Media was sterilized with 0.20 μm cellulose-nitrate filters. Cultural media were stored at 22°C prior the cell inoculation. SO_2_ was added immediately before the inoculum from a 4 g/L sodium metabisulphite in mQ water. The theoretical content of molecular SO_2_ was calculated according to Usseglio-Tomasset and Bosia (1984), Ribéreau-Gayon et al. (2006), and Duckitt (2012). Cellular growth was monitored by OD at 600 nm. Fresh cells in YPD broth were centrifuged at 3500 rpm for 15 min (Hettich, ROTINA 380R, Tuttlingen, Germany); then, cells were washed in 0.9% (w/v) NaCl and inoculated at 0.1 OD_600 nm_ in flask in SWM adjusted at 5% (v/v) ethanol, pH 4.5 and maintaining an air/medium ratio of at least 40% in order to ensure aerobic condition. Cellular pre-cultures were grown at 25°C for 3 days, in aerobic condition. An aliquot of the fresh cultures was analyzed by plate count to calculate the exact number of viable cells transferred into each variant of the SWM for the RSM (**Table 1**). The inoculum was carried out at 0.25 OD_600 nm_ in SWM modified as required by the RSM scheme (**Table 1**). The inoculated media were divided into 10 mL aliquots in sterile and hermetically closed tubes with no headspace volume, and cultivated at 22°C in static condition. Each aliquot sample was used once for analyses. Cellular growth was monitored daily by total plate count and OD_600nm_ measurement. At 1.00 ± 0.2 OD_600 nm_ cells two aliquots were pelleted by centrifugation (11000 rpm, 1 min, 4°C) (Hettich, ROTINA 380R, Tuttlingen, Germany), collecting a total cell amount of 20 OD_600 nm_, immediately frozen with liquid nitrogen and stored at -80°C until use. For the RSM scheme, the cultures were arranged according to the chosen experimental design.

**Table 1:** Runs of Box–Behnken experimental design, normalized relative expression values of *DbCD* and *DbVPR* genes, expressed as fold-change, and quantification of vinyl phenol, vinyl guaiacol, ethyl phenol, and ethyl guaiacol, expressed as ratios between μmoles of product (volatile phenols) on μmoles of relative consumed precursor (coumaric and ferulic acids) for the different trials.

						Conc. (mg/L)						Yield (μM product/μM consumed acid)			
							
Run	mol. SO_2_ (mg/L)	pH	Ethanol (v/v)	*DbCD*	*DbVPR*	*p*-cumaric acid	Ferulic acid	Vinyl phenol	Vinyl guaiacol	Ethyl phenol	Ethyl guaiacol	Vinyl phenol	Vinyl guaiacol	Ethyl phenol	Ethyl guaiacol
1	0	3.5	8.75	0.49	1.22	2.35	1.64	1.09	0.036	2.45	2.29	0.25	0.01	0.55	0.58
2	0.25	3.5	8.75	0.45	1.33	2.39	1.92	0.71	0.029	4.15	3.46	0.16	0.01	0.94	0.93
3	0	4.5	8.75	0.52	1.47	3.81	1.81	1.13	0	2.67	1.92	0.35	0.00	0.80	0.51
4	0.25	4.5	8.75	0.66	1.80	4.11	2.82	0.35	0	2.54	1.57	0.12	0.00	0.83	0.53
5	0	4	5	0.30	1.47	0.86	0.42	0.40	0	5.73	4.29	0.07	0.00	1.02	0.87
6	0.25	4	5	0.14	0.80	3.86	2.66	0.10	0	2.53	1.96	0.03	0.00	0.77	0.63
7	0	4	12.5	0.24	0.67	3.43	2.43	1.87	0.075	2.16	2.00	0.53	0.02	0.60	0.60
8	0.25	4	12.5	0.38	0.91	3.45	2.78	2.24	0.324	2.52	2.28	0.64	0.11	0.70	0.75
9	0.125	3.5	5	0.33	0.99	0.39	0.37	0.14	0	6.45	5.18	0.02	0.00	1.08	1.04
10	0.125	4.5	5	0.39	1.11	1.26	0.66	0.26	0	5.65	3.90	0.05	0.00	1.07	0.82
11	0.125	3.5	12.5	0.40	0.94	2.34	2.33	1.75	0.092	2.41	2.70	0.40	0.03	0.54	0.80
12	0.125	4.5	12.5	0.65	0.92	4.08	2.07	1.55	0.021	2.06	1.37	0.51	0.01	0.66	0.38
13	0.125	4	8.75	0.21	0.85	3.16	2.13	0.59	0	3.56	2.62	0.16	0.00	0.93	0.74
14	0.125	4	8.75	0.15	0.89	2.29	1.61	0.51	0.024	2.60	2.00	0.11	0.01	0.58	0.50
15	0.125	4	8.75	0.18	0.72	3.17	2.06	0.76	0	3.37	2.61	0.20	0.00	0.88	0.72


### Extraction of Total RNA and cDNA Synthesis

The extraction of total RNA from pellets was carried out using Presto Mini RNA Yeast Kit (Geneaid, New Taipei City, Taiwan) with few modifications. Briefly, cell lysis through mechanic disruption was performed in 500 μL Buffer RB, 5 μL β-mercaptoethanol, and an iso-volume of glass beads (425–600 μm, 154 Sigma–Aldrich, Saint Louis, MO, United States). Three breaking cycles with TissueLyser (Qiagen, Hilden, Germany) for 2 min at the maximum oscillation frequency, interchanged with 1 min on ice, were applied. The supernatant was centrifuged at 16000 × *g* for 3 min (Hettich, Tuttlingen, Germany). The genomic DNA residue was degraded using 100 μL of 2 KU/mL DNase (Sigma–Aldrich, St. Louis, MO, United States) for 15 min at room temperature. Following steps were carried out according to the manufacturing’s instructions. RNA concentration was determined by measuring the absorbance at 260 nm (BioTek, Winooski, VT, United States). The integrity of RNA sample (0.3 μg RNA, 2 μL RNA loading Buffer 5X, H_2_O DEPC up to 10 μL) was assessed, after 5 min treatment at 65°C, by electrophoresis on 1.2% agarose gel [90 mL DEPC water, 10 mL 10X formaldehyde gel buffer (200 mM MOPS, 50 mM sodium acetate, and 10 mM EDTA)] adjusted at 7 pH with NaOH prepared in DEPC water 37% (v/v) formaldehyde added. The electrophoretic run was carried out at 100 V for 1 h and bands were UV visualized (Bio-Rad, Berkeley, CA, United States). RNAs were stored at -80°C until cDNA synthesis. The RNA retrotranscription was obtained with the QuantiTect Reverse Transcription Kit (Qiagen, Hilden, Germany). cDNAs were stored at -20°C until used for the qPCR assays.

### Primer Design

Five genes, pyruvate decarboxylase (PDC) (*DbPDC*), aldehyde dehydrogenase (*DbALD*), actin (*DbACT*), eukaryotic translational elongation factor (EF) (*DbEF*), and tubulin (*DbTUB*), were analyzed to identify a HKG suitable in the normalization process of the gene expression of CD (*DbCD*) and VPR (*DbVPR*) (**Table 2**). Gene sequences of *DbPDC* and *DbALD* were identified using *S. cerevisiae* S288C (Schifferdecker et al., 2014), *Komagataella phaffii* CBS7435 and GS115, *D. bruxellensis* CBS2499 (Piškur et al., 2012), and *B. bruxellensis* AWRI1499 (Curtin C.D. et al., 2012) genomes. SGD^1^, NCBI^2^, and ENA^3^ databases were used as sequence sources. All alignments were performed through BLAST and ClustalIX2. Primer pairs were obtained at NCBI website^4^ and validated for no forming neither self nor cross-dimers^5^ (**Table 2**). The *DbCD* gene sequence for primer design was deduced by Godoy et al. (2014).

**Table 2:** Primer pairs used for quantitative PCR (qPCRs).

Oligo name	Sequence (5′ → 3′)	Tm (°C)	Reference
*DbALD*_F	CTATCAAGGTCGGAAACCCA	57.3	This study
*DbALD*_R	TCTCTCACCACCAGTAAGGA	57.3	This study
*DbACT*_F	TTATTGATAACGGTTCTGGTATGT	55.9	Nardi et al., 2010
*DbACT*_R	ACCCATACCGACCATGATAC	57.3	Nardi et al., 2010
*DbEF*_F	CTCCAGTTGTTGACTGCCA	56.7	Nardi et al., 2010
*DbEF*_R	CATCTTAACCATAGCAGCATCAC	58.9	Nardi et al., 2010
*DbPDC*_F	GTGGTTTGCTTTCCGACTAC	57.3	This study
*DbPDC*_R	AAACAGCGGACTTGACCTTAC	57.9	This study
*DbTUB*_F	GTATCTGCTACCAGAAACCAACC	60.6	Rozpędowska et al., 2011
*DbTUB*_R	CCCTCACTAACATACCAGTGGAC	62.4	Rozpędowska et al., 2011
*DbCD*_F	CACAGACTCGAACGGAAAAC	57.3	Godoy et al., 2014
*DbCD*_R	CCAGGGCGTACACATTGATA	57.3	Godoy et al., 2014
*DbVPR*_F	CTAAGGGCACTATCAAGGACA	57.9	Romano et al., 2017
*DbVPR*_R	CTGCAAAGAACCAGCATCA	54.5	Romano et al., 2017


### PCR Assays

Two sets of gene expression analysis were set up under different oenological conditions: (i) to identify a suitable HKG for gene expression normalization; (ii) to analyze the relative expression of *DbCD* and *DbVPR*, by using the gene identified in (i). As far the primers couples designed in this study for *DbCD*, *DbALD*, and *DbPDC* genes, they were also validated by a standard PCR amplification in a 25 μL reaction composed by: 1 U Taq, 200 μM dNTPs (Biotech rabbit, Dusseldorf, Germany), 1X Taq Buffer (Genscript, Piscataway, NJ, United States), 1 mM MgCl_2_ (5Prime, Hilden, Germany), 0.1 μM primer forward and 0.1 μM primer reverse (Eurofins Genomics, Ebersberg, Germany), and 80–100 ng DNA. The amplification cycle was: 95°C for 6 min, 95°C for 45 s/54°C for 30 s/72°C for 1 min (repeated 34 times), and 72°C for 10 min. Results were visualized on a 2% agarose gel prepared in TAE 1X buffer (20 mL TAE 50X, 980 mL demineralized water) and 0.5 μg/mL ethidium bromide. Electrophoresis was set at 80 V for 1.30 h. PCR products were sequenced by an external provider (Eurofins genomics, Ebersberg, Germany).

As far qPCRs, they were performed in a Realplex Mastercycler EP Gradient Thermocycler (Eppendorf, Hamburg, Germany) using a 15 μL reaction mix composed as follow: 2X SYBR Green Master-Mix (Biotech rabbit, Dusseldorf, Germany), 200 nM-100 nM-50 nM primer forward and primer reverse (Eurofins genomics, Ebersberg, Germany), and 10-fold dilution cDNA. The qPCR amplification cycle was set at 95°C for 30 s, 54°C for 30 s, and 65°C for 30 s; repeated for 40 times. At the end of the reaction (95°C for 15 s), a melt-curve was generated by increasing the temperature from 60 to 95°C, with a step at 0.5°C. All cDNAs were run as technical duplicates in a 96-well plate (Eppendorf, Hamburg, Germany). For each gene, three decimal serial dilutions at least were prepared into DNA LoBind tubes (Eppendorf, Hamburg, Germany) and stored at -20°C. The amplification curves were analyzed with Realplex software (Eppendorf, Hamburg, Germany).

The 2^-ΔΔ*C*_T_^ method was applied on the basis of Livak and Schmittgen (2001) to calculate the relative expression of *DbCD* and *DbVPR* respect the chosen HKG expression. Results were expressed as fold-changes whereas the expression value of the target gene (normalized against *DbTUB* expression) was expressed as increase or decrease respect to its expression in the calibrator (for equivalent amount of samples) corresponding to the growth condition “LS” [0 mg/L mol. SO_2_, pH 4.5 and 5% (v/v) ethanol] described in the paragraph “Gene Expression Stability.”

### Gene Expression Stability

The expression of *DbPDC*, *DbALD*, *DbACT*, *DbEF*, and *DbTUB* genes was evaluated setting up a qPCR multiplex assay under two different oenological conditions of the SWM called “low-” and “high-” stringent (LS and HS, respectively) growth conditions. In particular, the LS condition was characterize by 0 mg/L mol. SO_2_, pH 4.5 and 5% (v/v) ethanol while the HS condition by 0.25 mg/L mol. SO_2_, pH 3.5 and 12.5% (v/v) ethanol. Yeast cultures were prepared in duplicate; three RNA extractions and the following cDNA synthesis were performed from each independent culture.

GeNorm analysis (Vandesompele et al., 2002) (Genex software version 4.3.6, MultiD analyses, Gothenburg, Sweden) was used to determine the stability of gene expression (termed *M*-value), by analyzing each reference gene against the others in a pairwise variation that serially excludes the least stable gene (highest *M*-value) from the analysis. At the end, genes are ranked with an accepted cut-off value of 0.50 according to their expression stability. Normfinder algorithm (Genex software version 4.3.6, MultiD analyses, Gothenburg, Sweden) separates the variation into an intra-group and an inter-group contribution. The analysis is repeated without considering the groups and this, estimates a robust standard deviation (SD) for each gene. The accumulated standard deviation (Acc. SD) is a reliable indicator of the number of reference genes to be used. All the genes were analyzed in the same assay to reduce any further experimental variability.

### Experimental Design and Response Surface Methodology

In order to investigate the expression of *DbCD* and *DbVPR* genes and the production of VPs in oenological conditions a Box–Behnken experimental design and RSM were applied. SWM samples were formulated with different level % ethanol (v/v) (5 – 8.75 – 12.5), pH values (3.5 – 4.0 – 4.5), and molecular SO_2_ (mg/L) (0 – 0.125 – 0.25) (**Table 1**). The 15 trials provided by Box–Behnken experimental design were analyzed using Statgraphics Plus 5.1 software. The expression values of investigated genes were normalized with the HKG expression.

The fit of the model was evaluated by the linearity coefficient (R-squared). The regression approach was used to determine the effects produced by SO_2_, pH, and ethanol variables. The main effects (A, B, and C) and both the linear (AB, AC, and BC) and quadratic effects (AA, BB, and CC) were statistically validated by analysis of variance. To identify the most important factors, a standardized Pareto chart is drawn. In particular, each effect is converted to a t-statistic by dividing it by its standard error (data not shown). These standardized effects are then plotted in decreasing order of absolute magnitude. Statistically relevant effects with a *p*-value less than 0.05 (95% confidence level) were reported in a response surface graph where the three-dimensional surface is described by a second-order polynomial equation.

### Determination of VPs

The content of hydroxycinnamic acids, namely *p*-coumaric and ferulic acids, vinyl phenol, vinyl guaiacol, ethyl phenol, and ethyl guaiacol in the cultures of the 15 runs of Box–Behnken experimental design was assessed in the obtained samples by an Acquity HClass UPLC (Waters, Milford, MA, United States) system equipped with a photo diode array detector 2996 (Waters). Chromatographic separations were performed with a Kinetex C18 150 mm × 3 mm, 2.6 μm particle size, 100 Å pore size (Phenomenex, Torrance, CA, United States). Eluting solvents were (A) trifluoroacetic acid 0.05% (v/v) and (B) methanol. The gradient program was 0.1 min, 20% B; 0.1–2 min, 35% B; 2–14 min, 58.5% B. The separation run was followed by 7 min of column rinsing and conditioning. The flow rate was 0.5 mL/min and the column temperature was 28°C. The samples were filtered with PVDF 0.22 μm filter prior the injection. Calibration curves were obtained for *p*-coumaric and ferulic acids, vinyl phenol, vinyl guaiacol, ethyl phenol, and ethyl guaiacol concentrations in the range from 0.1 to 20 mg/L. Quantification was performed according to the external standard method. Data acquisition and processing were carried out by Empower 2 software (Waters) at 320, 280, and 260 nm for hydroxycinnamic acids, ethyl phenols, and vinyl phenols, respectively. Yield values of VPs were calculated as the molar ratio between each product (vinyl phenol, vinyl guaiacol, ethyl phenol, and ethyl guaiacol) and the corresponding hydroxycinnamic acid potentially used as substrate. Data were analyzed with Statgraphics Plus 5.1 using the RSM approach.

## Results

The aim of the study was to investigate the expression of *DbCD* and *DbVPR* genes and the production of VPs in a range of oenological conditions. To do that, we defined the experimental conditions at the realistic concentrations of some factors found in wines along with the requirement to have conditions compatible with cell growth. Different runs (**Table 1**) were performed to obtain gene expression values workable through a RSM approach under the tested conditions: SO_2_ levels ranged from 0 to 0.25 mg/L, pH varied between 3.5 and 4.5 units and ethanol concentrations between 5 and 12.5% (v/v).

### Identification of *DbPDC* and *DbALD* Genes in *D. bruxellensis*

*DbPDC* gene was identified in the scaffold 1 at 1700 bps (e_gw1.1.1485.1) of *D. bruxellensis* CBS2499 genome; in particular, the nucleotide sequence showed about 55% identity with the *S. cerevisiae* genes encoding for *PDC1*, *PDC5*, and *PDC6* (55.1, 55.8, and 55.5%, respectively). Due to the similar level of identity found among the three isoforms, *PDC1* sequence was chosen for a further investigation in the genome of *B. bruxellensis* AWRI1499. The nucleotide sequence with accession number “EIF49850.1” was identified as a possible homologous of *S. cerevisiae PDC* gene with an identity of 55% (identity of 96.9% with e_gw1.1.1485.1). In *K. phaffii* genome, the gene codifying for *KpPDC* showed two potential isoforms differently located in *K. phaffii* CBS7435 (chromosomes 3 and 4). Only the sequence on the chromosome 3 identified the homologous gene (identity of 100%) on the genome of the strain *K. phaffii* GS115, with accession number XM_002492352.1. Thus, this gene was aligned against *D. bruxellensis* CBS2499 and the sequence in the scaffold 1 (e_gw1.1.1485.1) was confirmed as the potential homologous gene of *KpPDC* (55.5%identity). In conclusion, the open reading frames represented by the accessions e_gw1.1.1485.1 and EIF49850.1 of *D. bruxellensis* CBS2499 and *B. bruxellensis* AWRI1499, respectively, were identified as the homologous genes of *ScPDC1* and *KpPDC*.

As regards *DbALD*, among the three genes (*ScALD3, ScALD2*, and *ScALD6*) encoding for the sequence of *ScALD6* of *S. cerevisiae* S288c genome led to the identification of a possible homologous gene in *D. bruxellensis* CBS2499 genome in the scaffold 4 at 1523 bps (e_gw1.4.403.1) with an identity of 55.6%. *ScALD6* sequence was also aligned against the genome of *B. bruxellensis* AWRI1499 and the resulting amino acid sequence with the accession number “EIF46557.1” showed an identity of 56% (99.4% identity with e_gw1.4.403.1). In *K. phaffii* genome, the gene encoding for *KpALD* was identified on different chromosomes; the nucleotide sequence in the scaffold 20 at 1496 bps (e_gw1.20.29.1) of the chromosome 3 of the strain CBS7435 showed the highest identity (67.8%) with both e_gw1.4.403.1 and EIF46557.1 open reading frames of *D. bruxellensis* CBS2499 and *B. bruxellensis* AWRI1499 genome, respectively. Thus, these last genes were used for primer design being considered the homologous genes of *ScALD6* and *KpALD*.

### Primer Validation in Standard PCRs and Optimization of qPCR Experiments

The primer pairs designed on *DbALD*, *DbPDC*, and *DbCD* were evaluated for their ability to produce a specific fragment through a standard PCR and further sequencing of the amplified products. A unique amplification product of 140 bps for all the three genes investigated was obtained (data not shown). This value corresponds to the expected product length on the base of the size (**Table 2**) of *in vitro* primers design. No aspecific products were detected and no amplification was observed with *S. cerevisiae* S288C and *K. phaffii* GS115 used as negative controls. Primer specificity was confirmed by sequencing with a 100% identity with the target sequences.

All primers designed for the amplification of the potential HKGs (*DbALD, DbPDC, DbEF, DbTUB*, and *DbACT*) and the target genes (*DbCD* and *DbVPR*) were validated to assess whether the qPCR reactions were really optimized. Five dilutions of cDNA samples obtained from cell culture of *D. bruxellensis* CBS2499 grown in SWM at LS condition [0 mg/L SO_2_, pH 4.5, 5% (v/v) ethanol] were tested to evaluate the ones containing from 10^3^ to 10^6^ copies of template that were able to give amplification curves between 30 and 20 *C*_T_ values, respectively. The obtained *C*_T_s values were relatively low and similar; the lowest one (about 13) was given by *DbEF* gene, while the highest (about 20) was obtained for *DbCD* gene, thus revealing similar expression levels among the amplified genes. Then, a standard curve was created to assess primer efficiency of both the target genes and potential HKGs, as well as to be used as “standard” within the normalization plate used for HKG identification by qPCR. The *R*^2^ values obtained for all primer pairs ranged from 0.980 to 0.999.

### Analysis of the Gene Expression Stability of Potential HKGs

Five genes were evaluated for this purpose (**Table 2**): two genes encoding for metabolic enzymes, PDC and acetaldehyde dehydrogenase (ALD), were chosen based on their important role on fermentative metabolism and on NAD(P)H supply. The three others, encoding for EF, tubulin (TUB), and actin (ACT), have been already used as HKG in other studies (Nardi et al., 2010; Rozpędowska et al., 2011; Moktaduzzaman et al., 2016).

*DbALD, DbPDC, DbEF, DbTUB*, and *DbACT* were analyzed by a qPCR multiplex assay to identify the reference gene with a constant expression level across the experimental conditions under study. Expression stability of potential HKG genes were assessed at the two extreme growth conditions of the used experimental design, LS [0 mg/L SO_2_, pH 4.5, 5% (v/v) ethanol] and HS [0.25 mg/L SO_2_, pH 3.5, 12.5% (v/v) ethanol]. The cultures showed a negligible lag phase reaching a similar final biomass (1.4–1.7 OD_600 nm_) in 8 days. The absolute quantification approach was employed to obtain the qPCR results from the assayed normalization plate. Thus, a direct comparison between *C*_T_s of each sample and *C*_T_s of the standards (corresponding to the transcript copy number of each serial dilution of the HKG candidates) was accomplished. Overall, genes presented *C*_T_s spanning from 11 to 20, with *DbEF* and *DbPDC* having the lower values (**Table 3**). C_T_ data were submitted to GeNorm (Vandesompele et al., 2002) and Normfinder analysis. Because of the elimination process, GeNorm algorithm cannot identify an optimum reference gene and ended up by suggesting a pair of genes having the best same *M*-value of 0.186, *DbACT* and *DbTUB* (**Table 3**). For a single gene discrimination, Normfinder was employed along with GeNorm algorithm. Since samples came from two different treatment groups, Normfinder algorithm separated the variation into an intra-group and an inter-group contribution. The analysis was then repeated without considering the groups and this allowed to estimate a robust SD; the lowest SD (0.0929) was assigned to *DbTUB* (**Table 3**). A minimal value of the accumulated standard deviation was a great indicator of the optimal number of reference genes to be used for normalization. The highest expression stability revealed by *DbTUB*, attributed by both the lowest *M*-value and the SD, identifying this gene as the HKG for this study.

**Table 3:** Candidate genes for their potential as housekeeping genes (HKGs).

Gene	*C*_T_ values								*M*-Value	Acc. SD
			
	LSA	LSA	LSB	LSB	HSA	HSA	HSB	HSB		
*DbALD*	19.51	19.77	20.10	20.12	19.05	18.62	18.6	18.5	0.373	0.2398
*DbPDC*	14.2	13.98	14.04	13.94	14.46	14.65	15.06	15.13	0.564	0.1523
*DbEF*	11.77	11.65	12.22	12.19	12.66	12.64	13.42	13.19	0.741	0.2762
*DbTUB*	16.71	16.83	16.86	16.49	16.89	17.04	17.28	17.34	0.186	0.0929
*DbACT*	17.58	17.23	17.27	17.53	18.17	18.13	18.01	17.75	0.186	0.1443


*The second row indicates the two tested conditions: LS, low stringent growth condition and HS, high stringent growth condition, performed in two independent replicates (A and B). From each replicate three mRNAs were extracted and analyzed in qPCR assays. *M*-value is calculated by the GeNorm analysis while Normfinder algorithm and GenEx software calculate the accumulated standard deviation (Acc. SD) that is the expected SD if multiple reference genes are used for normalization.*

### Effect of SO_2_, pH, and Ethanol on *DbCD* and *DbVPR* Gene Expression

Real-time qPCR assays were carried out to test all conditions of the experimental design in order to study the role of SO_2_, pH, and ethanol on *DbCD* and *DbVPR* genes expression. All the assays produced amplification curves in the range of the best sensitivity of the qPCR (20–30 *C*_T_ values) and a high reproducibility within a single test and among tests was obtained; indeed, an overlapping of the amplification curves of the replicates of both each run and the calibrator was observed. This was particularly evident in the case of *DbTUB* amplification that showed a constant gene expression (*C*_T_ value of 23) among the 15 conditions evaluated, confirming once again its reliable role as HKG.

**FIGURE 1:**
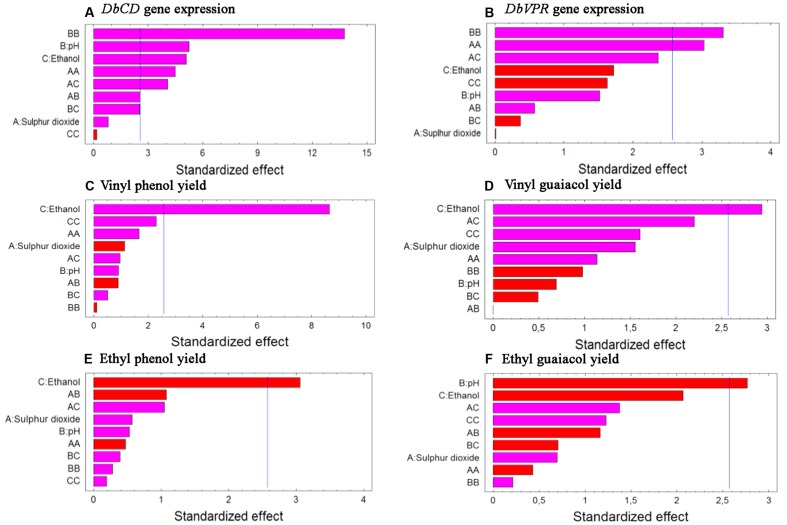
Standardized Pareto charts for each analyzed variable **(A)** *DbCD* and **(B)** *DbVPR* gene expression, **(C)** vinyl phenol, **(D)** vinyl guaiacol, **(E)** ethyl phenol, and **(F)** ethyl guaiacol yields. The color of the bars shows whether an effect is positive (pink) or negative (red). A line is drawn on the chart beyond which an effect is statistically significant at the specified significance level of 5%.

**FIGURE 2:**
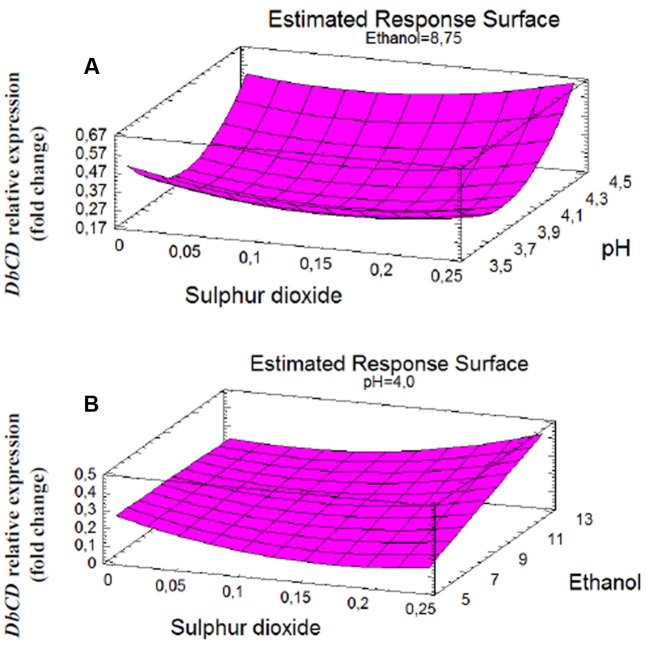
Response surface fitted to experimental data points corresponding to: **(A)** *DbCD* expression as function of SO_2_ and pH interaction (AB); **(B)** *DbCD* expression as function of SO_2_ and ethanol interaction (AC).

Although the experimental design has to be considered functional to only apply the RSM approach and data cannot be individually interpreted as not obtained from biological replicates (except for runs 13, 14, and 15), it was possible to observe that *DbCD* gene was downregulated in all the tested conditions with fold-change values ranging between 0.14 and 0.66 (**Table 1**). The application of the Box–Behnken results to the RSM approach allowed to analyze how the *DbCD* gene expression was influenced by SO_2_, pH, and ethanol by predicting further expression values inside the environment of the tested variables. Indeed, as regards the *DbCD* gene expression, a high R-squared values indicated a good fit of the model to the experimental data explaining the 98.3% (R-squared) of the *DbCD* gene variability (**Table 4**). Main and interaction effects (linear and quadratic) of the factors on the gene expressions are reported in **Table 5** and shown in the standardized Pareto chart (**Figure 1**). While pH and ethanol factors produced a significant effect (*P*-value < 0.01) on the *DbCD* gene expression, SO_2_ did not affect it. On the contrary, linear interactions between SO_2_ and pH and SO_2_ and ethanol revealed a substantial influence (*P*-value < 0.05) (**Table 5** and **Figure 1**) thus concurring to define the response represented as three-dimensional surface (**Figures 2A,B**).

**Table 4:** Regression equations which fitted to the data of the Box–Behnken experimental design.

Variable (*y*)	Regression model equation	*R*^2^ (%)
*DbCD* gene	*y* = 17.797 - 5.704^∗^A - 8.5935^∗^B - 0.0976963^∗^C + 5.47467^∗^AA + 0.756^∗^AB + 0.159467^∗^AC + 1.05217^∗^BB + 0.0249333^∗^BC - 0.000245926^∗^CC	98.3
*DbVPR* gene	*y* = 21.2275 - 12.6293^∗^A - 10.3565^∗^B + 0.186785^∗^C + 19.4453^∗^AA + 0.88^∗^AB + 0.4864^∗^AC + 1.32733^∗^BB - 0.0189333^∗^BC - 0.011603^∗^CC	87.3
Vinyl phenol yield	*y* = -0.10787 + 0.203333^∗^A + 0.173333^∗^B - 0.10463^∗^C + 4.34667^∗^AA - 0.56^∗^AB + 0.08^∗^AC - 0.0183333^∗^BB + 0.0106667^∗^BC + 0.00660741^∗^CC	94.5
Vinyl guaiacol yield	*y* = -0.619907 - 0.523333^∗^A + 0.346667^∗^B - 0.0109259^∗^C + 0.773333^∗^AA + 0.0^∗^AB + 0.048^∗^AC - 0.0416667^∗^BB - 0.00266667^∗^BC + 0.00121481^∗^CC	81.2
Ethyl phenol yield	*y* = 2.62032 + 5.05^∗^A - 0.6825^∗^B - 0.161407^∗^C - 2.61333^∗^AA - 1.44^∗^AB + 0.186667^∗^AC + 0.0966667^∗^BB + 0.0173333^∗^BC + 0.00118519^∗^CC	71.8
Ethyl guaiacol yield	*y* = 2.08079 + 4.24667^∗^A - 0.385833^∗^B - 0.060037^∗^C - 2.02667^∗^AA - 1.32^∗^AB + 0.208^∗^AC + 0.0633333^∗^BB - 0.0266667^∗^BC + 0.00645926^∗^CC	78.2


*Factors are mol SO_*2*_ (A), pH (B), and Ethanol (C). The second-order equations show main (A, B, and C), linear (AB, AC, and BC), and quadratic effects (AA, BB and CC). Coefficients are the regression coefficients for the considered variable. R-squared statistic indicates that the model as fitted explains a certain % of the variability in the considered variable.*

**Table 5:** Statistical analysis (value are expressed as *P*) of main effect of three variables and their interaction for *DbCD* and *DbVPR* expression levels and volatile phenol productions.

			Yield (μM product/μM consumed acid)			
			
Factor	*DbCD* gene	*DbVPR* gene	Vinyl phenol	Vinyl guaiacol	Ethyl phenol	Ethyl guaiacol
**Mol SO_2_ (A)**	0.456	0.989	0.3090	0.1805	0.5915	0.5164
**pH (B)**	**0.003**	0.190	0.4067	0.5201	0.6185	**0.0395**
**Ethanol (C)**	**0.004**	0.146	**0.0003**	**0.0323**	**0.0283**	0.0934
**AA**	**0.006**	**0.029**	0.1556	0.3078	0.6576	0.6859
**BB**	**0.000**	**0.021**	0.9146	0.3727	0.7917	0.8387
**CC**	0.8635	0.164	0.0710	0.1694	0.8552	0.2733
**AB**	**0.050**	0.593	0.4111	1.0000	0.3294	0.2973
**AC**	**0.010**	0.064	0.3810	0.0791	0.3417	0.2277
**BC**	0.052	0.727	0.6303	0.6456	0.7126	0.5124


*Bold values are those considered statistically significant (*P* < 0.005).*

**FIGURE 3:**
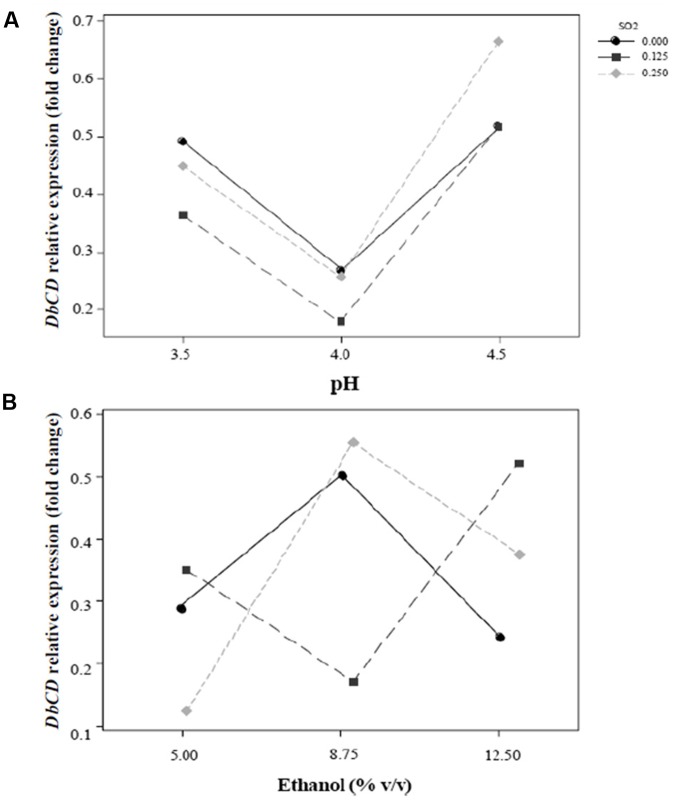
Interaction plots for the expression of *DbCD* gene. **(A)** SO_2_ and pH (AB); **(B)** SO_2_ and ethanol concentration (AC). Lines represent the predicted responses at further experimental combinations among the analyzed factors. Continuous line (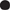), 0 mg/L mol. SO_2_; long-dashed line (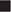), 0.125 mg/L mol. SO_2_; short-dashed line (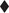), 0.25 mg/L mol. SO_2_.

The shape of the surface obtained for SO_2_ and pH interaction (**Figure 2A**) on the response reflected the predominant inhibition by pH, since the expression of the gene decrease rapidly up to pH 4. In particular, the change in *DbCD* expression occurring from the lowest to the highest level of pH (**Figure 3A**) was the same for both 0.125 and 0.250 mg/L levels of SO_2_; the parallel trend of lines indicated that the effect of the pH on the response is probably not dependent from these SO_2_ values. Even when pH was in the range 3.5–4 and SO_2_ at 0 mg/L, the observed lines were almost parallel with respect to the other lines (with an overlapping between 0 and 0.250 mg/L of SO_2_). On the contrary, when pH was set between 4 and 4.5 a moderate interaction of this factor with SO_2_ occurred (lines are not parallel) (**Figure 3A**).

On the other hand, the interaction between SO_2_ and ethanol produced a response that changed faster as function of ethanol (**Figure 2B**). In detail, considering ethanol from 5 to 12.5% (v/v) and SO_2_ at the concentration of 0 mg/L, 0.125 mg/L or 0.250 mg/L, the observed lines were not parallel indicating that an interaction between ethanol and SO_2_ exists (**Figure 3B**). If ethanol at 5% (v/v) interacted with 0.25 mg/L SO_2_, the *DbCD* expression was lower than the one revealed by the condition at 0 mg/L SO_2_. This is probably due to the effect of ethanol along with SO_2_ in determining more stress to the cell. Moreover, the expression at 0 mg/L SO_2_ and 8.75% (v/v) ethanol was slightly lower than the one revealed at 0 mg/L SO_2_ and 5% (v/v) ethanol.

The comparison between the two interaction plots (**Figures 3A,B**) allowed identifying the SO_2_-ethanol as the stronger interaction to define the expression of *DbCD*, as also showed by the *p*-value of this linear interaction (AC, **Table 5** and **Figure 1**).

Finally, based on the response surfaces for *DbCD* gene expression and the model equation it is also possible to predict further responses in addition to those obtained in this study; according to this prediction approach, the combination of the factor levels that maximizes the *DbCD* expression (0.834-fold change) is at 0.25 mg/L, 4.5 and 12.5% (v/v), respectively, for SO_2_, pH, and ethanol.

As far the *DbVPR* gene expression, it showed a different trend in regulation in comparison to *DbCD* gene. Even if data of the experimental design cannot be singularly interpreted, *DbVPR* seemed to be upregulated in runs 1, 2, 3, 4, 5, and 10 with fold-change value ranging between 1.11 and 1.80, whereas in the other cases it was slight downregulated, being values lower than 1. Interestingly, following the results in **Table 1**, although *DbCD* and *DbVPR* genes were expressed at their maximum level under the same growth condition corresponding to SO_2_ 0.25 mg/L, pH 4.5, ethanol 8.75% (v/v) (run 4). The statistical processing of expression data provided a regression equation of the proposed model with a goodness of fit of 87.3% (R-squared) (**Table 4**). In this case only a positive quadratic effect of SO_2_ and pH resulted statistically significant on the *DbVPR* gene expression being all the other factors, main and interactions, characterized by *p*-values higher than 0.05 (**Table 5**). In agreement with the RSM approach, *DbVPR* expression was maximizes (1.80-fold change) at 0.25 mg/L SO_2_, pH 4.5, and 12.5% (v/v) ethanol, as observed for the *DbCD*.

### Effect of SO_2_, pH, and Ethanol on VP Production

The release of VPs was determined in the experimental conditions adopted in the Box–Behnken experimental design. Although 10 mg/L of each hydroxycinnamic acid were added to the SWM, the initial concentrations of *p*-coumaric acid and ferulic acid were estimated at 8.40 ± 0.07 mg/L and 6.71 ± 0.25 mg/L, respectively. As expected, these compounds proportionally decreased as the VPs increased (data not shown). The highest concentration of VPs was reached under a condition that is more permissive the yeast growth [SO_2_ 0.125 mg/L, pH 3.5 and ethanol 5% (v/v)] in comparison to the expression of *DbCD* and *DbVPR* genes [SO_2_ 0.25 mg/L, pH 4.5, and ethanol 12.5% (v/v)]. Indeed, VPs are released at a final concentration of 6.45 and 5.18 mg/L of ethyl phenol and ethyl guaiacol, respectively, in run 9 whereas *DbCD* and *DbVPR* genes were approximatively half of the expression values detected in run 4.

In general, some considerations arose from the calculated yields of VPs (**Table 1**). First, the lowest conversion of acids in the corresponding vinyl compounds was detected for the vinyl guaiacol that was mostly produced at trace level in all the analyzed runs (**Table 1**). We could speculate that this behavior could be linked to a higher activity of *DbCDp* toward the coumaric acid rather than the ferulic acid. On the contrary, ethyl phenol and ethyl guaiacol yields were found relatively balanced each other suggesting a similar capability of the *DbVPR* enzyme to transform its two substrates, the vinyl derivates. However, for this observation studies are required to analyze the activity of *DbCDp* and *DbVPRp* in the metabolic pathway of VPs under enological conditions.

Data processing by the RSM approach released four second-order equations with R-squared values indicating that the model as fitted explained 94.5, 81.2, 71.8, and 78.2% of the variability in vinyl phenol, vinyl guaiacol, ethyl phenol, and ethyl guaiacol molar ratios, respectively (calculated against the corresponding substrates of hydroxycinnamic acids). Main and interaction effects (linear and quadratic) of the factors on the VP production are reported in **Table 5** and shown in the standardized Pareto charts (**Figure 1**). Considering the influence of individual factors, ethanol, and pH produced a significant effect (*P*-value < 0.05) on the production of such aromatic compounds whereas SO_2_ did not result involved in. In particular, ethanol influenced the release of vinyl phenol, ethyl phenol, and vinyl guaiacol while pH was important in determining the variability of ethyl guaiacol. No linear interaction between factors resulted statistically significant for the synthesis of VPs.

## Discussion

Wine spoilage by *D./B. bruxellensis* has increased in frequency because of the use of less-severe processing conditions, the great variety of diverse vinification techniques and the tendency to reduce the use of preservatives, such as sulfur dioxide. In particular, the sustainable perspective that to limit SO_2_ in bottled wines can reduce undesirable allergenic effects on humans drives the latter action.

The capability of *D./B. bruxellensis* to survive and to grow in wine can be partially ascribed to its high resistance to SO_2_; one of the main research question that can be addressed regarding the prevention of this spoilage yeast species is: “how the SO_2_ addition can be managed in order to counteract the yeast occurrence during winemaking and in the final product?” Unfortunately, since the active form of SO_2_ against microbial proliferation depends on pH, ethanol concentration, and temperature (Usseglio-Tomasset and Bosia, 1984; Ribéreau-Gayon et al., 2006), the answer has to take into consideration that wine is an extremely heterogeneous environment.

Although some wine factors/constituents are reported to play a key role on the off-flavor synthesis by *D./B. bruxellensis*, most of the works carried out to date have independently studied the factors without considering their interactions (Dias et al., 2003b; Godoy et al., 2008; Sturm et al., 2014). With the RSM approach used in this study, the simultaneous effects produced by SO_2_, pH, and ethanol on *DbCD* and *DbVPR* gene expression and VPs production have been investigated. Two specific aims are issued in this investigation: (i) the identification of a suitable HKG to assess the relative expression of *DbCD* and *DbVPR* genes and (ii) the setup of an experimental design in order to predict factors and/or possible factor interactions affecting the pathway of VP production.

Regarding the first goal, since real-time qPCR represents the protocol for highly sensitive and reproducible gene expression analysis, accurate and reliable expression results cannot exclude the normalization of real-time qPCR data against a “confident” reference gene in the condition under study. In this work, five genes were evaluated for this purpose and the GeNorm and Normfinder algorithm were used to assay the RNA transcription level of each candidate gene. Despite to the large literature reporting real-time qPCR expression data of several *D./B. bruxellensis* genes, only one manuscript has searched for adequate HKGs to be involved in the data normalization of gene expression assays under oenological conditions (Nardi et al., 2010). In particular, Nardi et al. (2010) choose actin (*ACT1*) and translational elongation factor EF-1α (*TEF1*) genes as housekeeping references. The finding that tubulin (*DbTUB*) was the best reference gene in the present study proves the need of include, as a specific objective of the work, preliminary transcriptional assays to validate the “housekeeping” status of a candidate reference gene under particular experimental conditions.

As concern the second goal, different considerations can be done on the analysis of possible factors that influence the expression of *DbCD* and *DbVPR* genes and the production of VPs.

In general, the main outcome of this study reveals that the highest variability of the response, as a function of the studied factors, was obtained with the expression of *DbCD* that resulted repressed in all the conditions tested by the experimental design in comparison with the condition used as “calibrator.” Indeed, being the first enzyme of the metabolic pathway of VPs, the *DbCD* gene is probably more influenced by change of the environmental/oenological conditions in comparison to the *DbVPR* gene.

The expression of *DbCD* is strongly affected by pH and the linear interactions between pH and SO_2_, SO_2_ and ethanol. Regarding the effect exerted by pH on *DbCD* expression, is important to consider that pH plays an important role on the enzyme substrates, determining the dissociation/undissociation of hydroxycinnamic acids. At wine pH both *p*-coumaric and ferulic acids are mainly under undissociated form (pKa = 4.5), that, due to their lipophilic properties, easily cross the periplasmic membrane and decrease cytoplasmic pH by dissociation into cytosol (Agnolucci et al., 2010). This means that in our study, *DbCD* expression would be expected to increase in the entire range of pH 3.5–4.5, and not only from pH 4 to 4.5, in order to convert acids into the corresponding vinyls. Interestingly, the maximal downregulation can be observed under conditions of pH 4. A hypothesis of this behavior of *DbCD* expression could be related to different mechanisms of the hydroxycinnamic acids uptake in *D. bruxellensis* CBS2499, by passive as well as by active transport, which would deserve more detailed analysis. However, we cannot also exclude the possibility of a strong downregulation resulting from the presence of higher level of SO_2_ at low pH.

Although it has been suggested that the entry of the hydroxycinnamic acids into cells is facilitated by the localization of ethanol close to the dehydrated membrane (Sousa et al., 1996), a high ethanol concentration can generate a cessation of the *DbCD* enzyme activity reducing the conversion of the hydroxycinnamic acids into vinyl phenols (Benito et al., 2009). Moreover, ethanol can also determine a post-transcriptional regulation of the CD affecting the protein activity (Clausen et al., 1994; Cavin et al., 1998). Thus, the same effect that ethanol produces on the membrane permeability is possibly the same exerted on enzyme’s conformation since this last depends mainly on the hydrophobic interactions among the amino acid residues of the protein (post-transcriptional regulation). We could speculate that the relative lower level of downregulation of *DbCD* gene observed in cells growing in presence of higher concentration of ethanol [0.25 mg/L, 4.5 and 12.5% (v/v)] could allow the cells compensating, by a transcriptional regulation of *DbCD* gene, a decreased enzyme activity.

Neither a main nor an interaction effect seem to influence *DbVPR* gene expression in the growth conditions under our study. However, the quadratic effect of pH and SO_2_ show a significant role in its expression. Indeed, under oenological conditions, SO_2_ causes undoubtedly oxidative stress, and we cannot forget that VPR enzyme has been identified in *D. bruxellensis* CBS4481 as a Zn/Cu superoxide dismutase (SOD1) (Granato et al., 2014).

The present study shows that the observed production of VPs, in the tested conditions, depends mainly on ethanol, as single factor, although pH is important in modulating the ethyl guaiacol yield. Moreover, a higher gene expression (run 4, **Table 1**) did not lead to a higher release of VPs (run 9, **Table 1**). This finding suggests that the transformation yield could be affected by factors other than *DbCD* and *DbVPR* regulation.

Ethanol plays a positive linear effect in the transformation of hydroxycinnamic acids to vinyl derivates. This result can support the finding that a lower downregulation of the *DbCD* gene occurs at a high ethanol concentration when cells have to counteract a possible lost in enzyme conformation. Contrarily to what has been observed by Chandra et al. (2014), the SO_2_ factor seems to have no effect on the effective production of ethyl phenols, and in general on the off-flavor yields. Nevertheless, different wines and winemaking procedures can affect the content of this chemical and, usually, a higher level is reached during aging, due to a mismanaging use of SO_2_ by oenologists. Further experiments are so required to investigate the pathway of VPs by *D./B. bruxellensis* in real wines or under more severe conditions. Finally, due to a diverse capability to counteract the SO_2_ stress, different *D./B. bruxellensis* strains could behave differently (Curtin C. et al., 2012; Vigentini et al., 2013); however, this work suggests that the uncontrolled use of sulfur dioxide, besides not representing a sustainable choice, may not be an adequate strategy to protect wine from spoilage.

## Author Contributions

FV contributed to the design of the work, to the selection of candidate genes for the normalization of gene expression, to perform the qPCR assays and to the interpretation of data for the work, to draft the work and revising it. DF contributed to analysis of volatile phenols, to the interpretation of data for the work and to draft the work. AC contributed to the preparation of cell cultures for the RMS approach, to the extraction of RNAs and the preparation of cDNA, to perform the qPCR assays and to draft the work. CaC contributed to the setup of qPCR assays. RF and CoC contributed to the interpretation of data for the work and to draft the work. IV contributed to the design of the work, to the acquisition, the analysis, and the interpretation of data for the work, to draft the work and revising it for important intellectual content, and ensured that that questions related to the accuracy or integrity of any part of the work were appropriately investigated and resolved.

## Conflict of Interest Statement

The authors declare that the research was conducted in the absence of any commercial or financial relationships that could be construed as a potential conflict of interest.

## Footnotes

1 http://www.yeastgenome.org

2 https://www.ncbi.nlm.nih.gov

3 http://www.ebi.ac.uk/ena

4 https://www.ncbi.nlm.nih.gov/tools/primer-blast

5 https://www.thermofisher.com
